# Grading criteria for venous invasion in thoracic esophageal squamous cell carcinoma

**DOI:** 10.1186/s13019-023-02272-8

**Published:** 2023-04-07

**Authors:** An Wang, Xiaojia Liu, Lu Lu, Shaohua Wang, Xiaofeng Chen

**Affiliations:** 1 Department of Thoracic Surgery, Huashan Hospital, Fudan University, Shanghai, China; 2 Department of Pathology, Huashan Hospital, Fudan University, Shanghai, China

**Keywords:** Esophageal squamous cell carcinoma, Venous invasion, Prognosis

## Abstract

**Background:**

Venous invasion (VI) is an adverse prognostic indicator in esophageal squamous cell carcinoma. However, grading criteria for venous invasion in thoracic esophageal squamous cell carcinoma (ESCC) have not been established.

**Methods:**

We enrolled 598 thoracic ESCC patients from 2005 to 2017. We detected the presence of venous invasion using the hematoxylin and eosin (H&E)-staining method and evaluated the VI grade on the basis of the number and maximal size of the involved veins. The degree of VI was classified as either 0, V1, V2, or V3, according to the combination of V-number and V-size.

**Results:**

The 1-year, 3-year and 5-year disease-free survival rates were 79.7%, 64.7% and 61.2%, respectively. Multivariate analysis demonstrated that lymphatic invasion (HR: 1.457, 95% CI: 1.058–2.006, p = 0.021), T category (HR: 1.457, 95% CI: 1.058–2.006, p = 0.022), N category (HR: 1.535, 95% CI: 1.276–2.846, p < 0.001), stage (HR: 1.563, 95% CI: 1.235–1.976, p < 0.001) and the degree of venous invasion (HR: 1.526, 95% CI: 1.279–2.822, p < 0.001) were significant indicators of recurrence. The disease-free survival curves were distinguished especially well by the degree of venous invasion in stage III and IV patients.

**Conclusions:**

The present study explored an objective grading criterion for VI and proved the prognostic value of the degree of venous invasion in ESCC. The classification of venous invasion into 4 groups is useful for the differentiation of prognosis in ESCC patients. The prognostic significance of the degree of VI in advanced ESCC patients for recurrence may have to be considered.

## Introduction

Esophageal cancer is the 9th most common carcinoma, and the mortality rate is 6th among all cancers [1]. The treatment of esophageal cancer involves a multidisciplinary comprehensive treatment model that includes surgery, radiotherapy and chemotherapy, endoscopic therapy, and immunotherapy [2]. The overall 5-year survival rate is 15–25% for esophageal cancer patients due to the advanced stage when they are diagnosed, and the 5-year survival rate for patients with resectable tumors is only 35-45% after surgery [3]. Venous invasion is a key prognostic indicator for esophageal squamous cell carcinoma (ESCC) [4].

Lymphatic (small vessel) invasion should be differentiated from venous (vascular/large vessel) invasion in esophageal cancer patients because the type of invasion may indicate a difference in prognosis on the basis of the 8th AJCC Cancer Staging Manual [5, 6]. Venous invasion was characterized as malignant tumor cells wandering in the sized veins [7]. Some studies have focused on the association between VI and prognosis in esophageal cancer [6, 8, 9]. In Japan, according to the Japan Esophageal Society, venous invasion is classified into four groups [V0 (none), V1 (slight), V2 (moderate), V3 (severe)] [10]. However, detailed information on this classification has not been documented. Venous invasion can be divided into intramural venous invasion (IMVI), which is limited to the mucosa, submucosa, muscularis propria, and extramural venous invasion (EMVI), which is limited to the adventitia microscopically [6]. We aim to establish objective criteria for the grading of VI and investigate its role in the prognosis of ESCC.

## Method

### Patients

The computerized and manual searches were performed with the keywords ‘Ivor-Lewis esophagectomy’ OR ‘McKeown esophagectomy’ AND ‘R0 resection’ AND ‘thoracic esophageal squamous carcinoma’ in our hospital database. Exclusion criteria were as follows: patients were confirmed as having “carcinoma in situ”; patients received preoperative neoadjuvant chemotherapy and/or radiotherapy. Finally, a total of 598 thoracic ESCC patients from 2005 to 2017 were included in the present study. Seventeen patients underwent the Ivor-Lewis procedure, and 581 patients underwent the McKeown procedure. All patients underwent at least mediastinal (including lymph nodes along bilateral recurrent laryngeal nerves) and abdominal lymph node dissection.

Preoperative examinations included cardiopulmonary function evaluation, computed tomography of the neck, chest and abdomen, endoscopy, and esophagography. Some patients received endoscopic ultrasound and positron emission tomography in recent years. The postoperative in-hospital mortality was 1.3% (8 of 598). The number of dissected lymph nodes ranged from 8 to 90, and the median was 39. The median number of positive lymph nodes was 1 (0–28). Chemotherapy with 5-fluorouracil and cisplatin was used to reduce the risk of recurrence after surgery in 95 advanced-stage patients. The present study was approved by the Research Ethics Board, and written individual informed consent was waived.

### Data collection

Clinical characteristics such as age, sex, tumor location, histological differentiation, pathological T parameter, pathological N parameter, pathological M parameter (supraclavicular lymph node metastasis), and pathological disease stage were collected from our hospital database. The seventh edition of the UICC TNM staging system was used to assess the pathological stage of the tumor.

### Histopathologic examination of VI

**Fig. 1:**
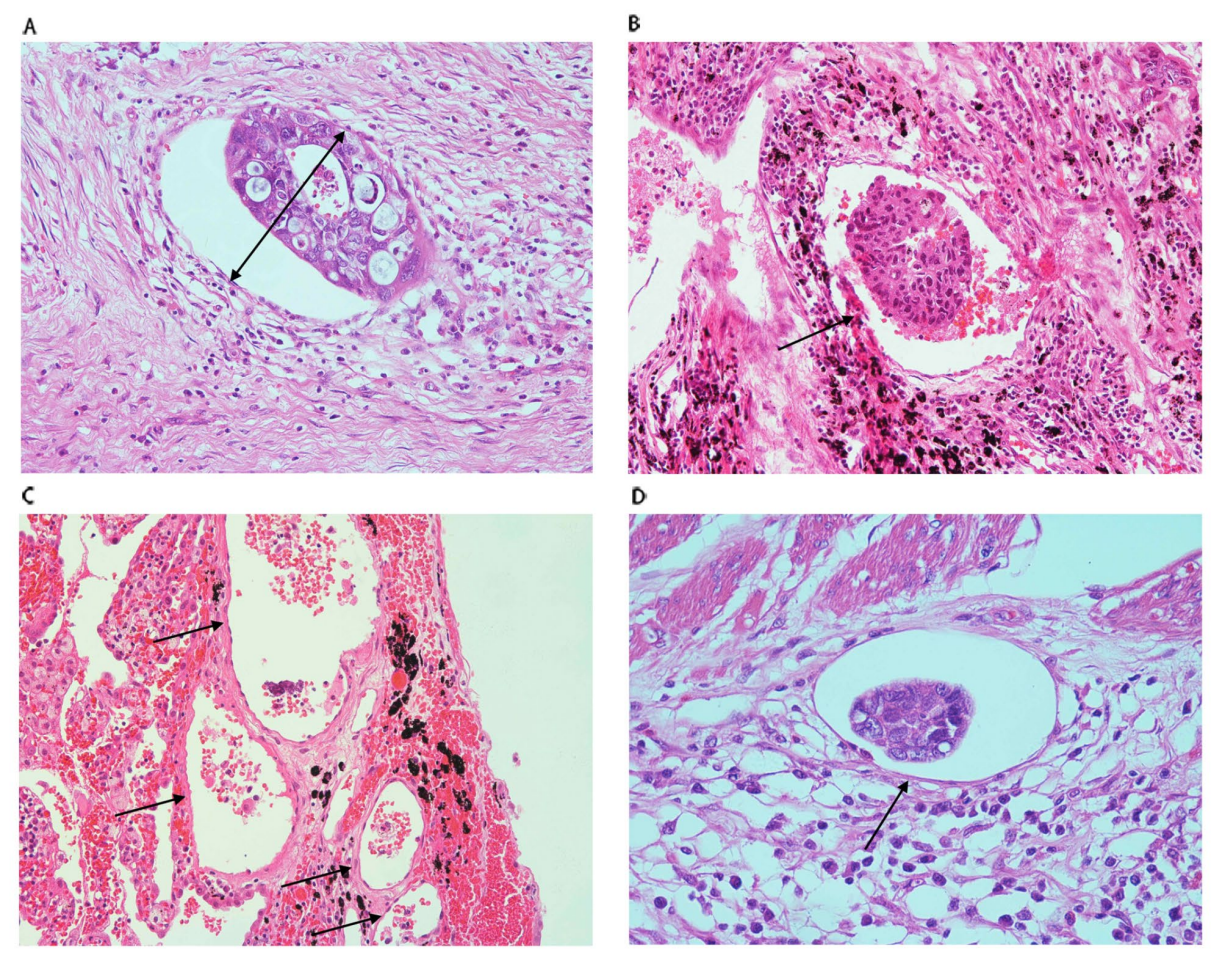
VI refers to tumor cells permeating into blood vessels. A: Example of VI indicating the maximal size (arrow) of VI. B: Example of VI showing the number of VI (arrow) (1–3); C: Example of VI showing the number of VI (arrow) (≥ 4). D: Example of extramural venous invasion (arrows)

Two gastrointestinal pathologists were enrolled in the assessment of venous invasion on H&E-stained tumor slides for each patient (Fig. 1A). We evaluated the VI grade on the basis of the number and maximal size of the involved veins. The method for assessing the number of VIs involved dividing the total number of VIs in all sections examined by the number of observed sections on every glass slide. The measure of VI number was classified into 2 grades: 1–3 and ≥ 4 (Fig. 1B and C). The maximal size of the VI was also taken into consideration on the basis of the minor axis of the largest vascular vessel on the glass slide (Fig. 1A). V-size was classified into V(s)-low < 1 mm and V(s)-high ≥ 1 mm. The degree of VI was classified into 0, V1, V2, and V3 according to the combination of V-number and V-size. EMVI was an independent poor prognostic parameter for esophageal cancer after surgery [11]. The depth of EMVI was deep. So, EMVI (Fig. 1D) was classified as V3 regardless of V-number and V-size. The detailed classification of VI was based on the V-number and V-size (Table 1). Additionally, V1, V2 and V3 could be presented in Fig. 1B, A and C respectively.

**Table 1:** Classification of VI

	V0 (None)	V1 (Slight)	V2 (Moderate)		V3 (Severe)
V-number	0	1–3	1–3,	4	≥ 4
V-size (mm)	0	< 1	≥ 1	< 1	≥ 1
EMVI	Absent	Absent	Absent	Absent	Present^*^

*: any V-number and V-size

**Table 2:** Demographic characteristics and clinicopathologic variables

	Venous invasion (N)				P value
	(V0)	(V1)	(V2)	(V3)	
Age					0.702
< 65	189	94	24	7	
≥ 65	169	88	24	3	
Gender					0.313
Male	307	147	43	9	
Female	51	35	5	1	
Tumor location					0.166
Upper	48	16	3	3	
Middle	179	86	26	3	
Lower	131	80	19	4	
Differentiation					0.120
Well	112	57	8	2	
Moderate	117	71	20	2	
Poor	129	54	20	6	
Lymphatic invasion					< 0.001
Absence	247	77	20	2	
Presence	111	105	28	8	
T category					< 0.001
T1	189	34	3	2	
T2	42	20	4	1	
T3	127	125	41	6	
T4	0	3	0	1	
N category					< 0.001
N0	169	43	6	1	
N1	115	59	15	2	
N2	55	57	18	4	
N3	19	23	9	3	
TNM Stage					< 0.001
I	128	17	2	1	
II	102	45	7	1	
III	97	98	33	6	
IV	31	22	6	2	

### Statistics

Fisher’s exact test was adopted to compare the clinical characteristics of patients with and without VI, and the log-rank test was used for univariable survival analyses of disease-free survival (DFS). DFS was defined as the date from surgery to the time of first diagnosed relapse or death. The prognostic significance of clinical indicators was examined in univariable analyses. The Kaplan–Meier method was used to calculate survival curves. Patients were censored at the last point of follow-up without contact. Clinical variables with a p value < 0.1 in univariable analysis were included in the multivariable analysis. A Cox proportional hazards model for multivariable analysis was used to delineate significant prognostic factors for DFS. Hazard ratios (HR) with 95% confidence intervals (CI) were generated in multivariable analysis. For all statistical analyses, a p value < 0.05 was considered statistically significant. Statistical analysis was performed using SPSS 19.0 software (IBM Corporation, Armonk, NY, USA).

## Results

### Detailed information of VI

The number of VI + patients was 240. The rate of VI positive was 40.1% (240/598). A total of 240 VI patients could be divided into V1 (182), V2(48), V3(10).

### Disease-free survival and univariate and multivariate analyses

**Fig. 2:**
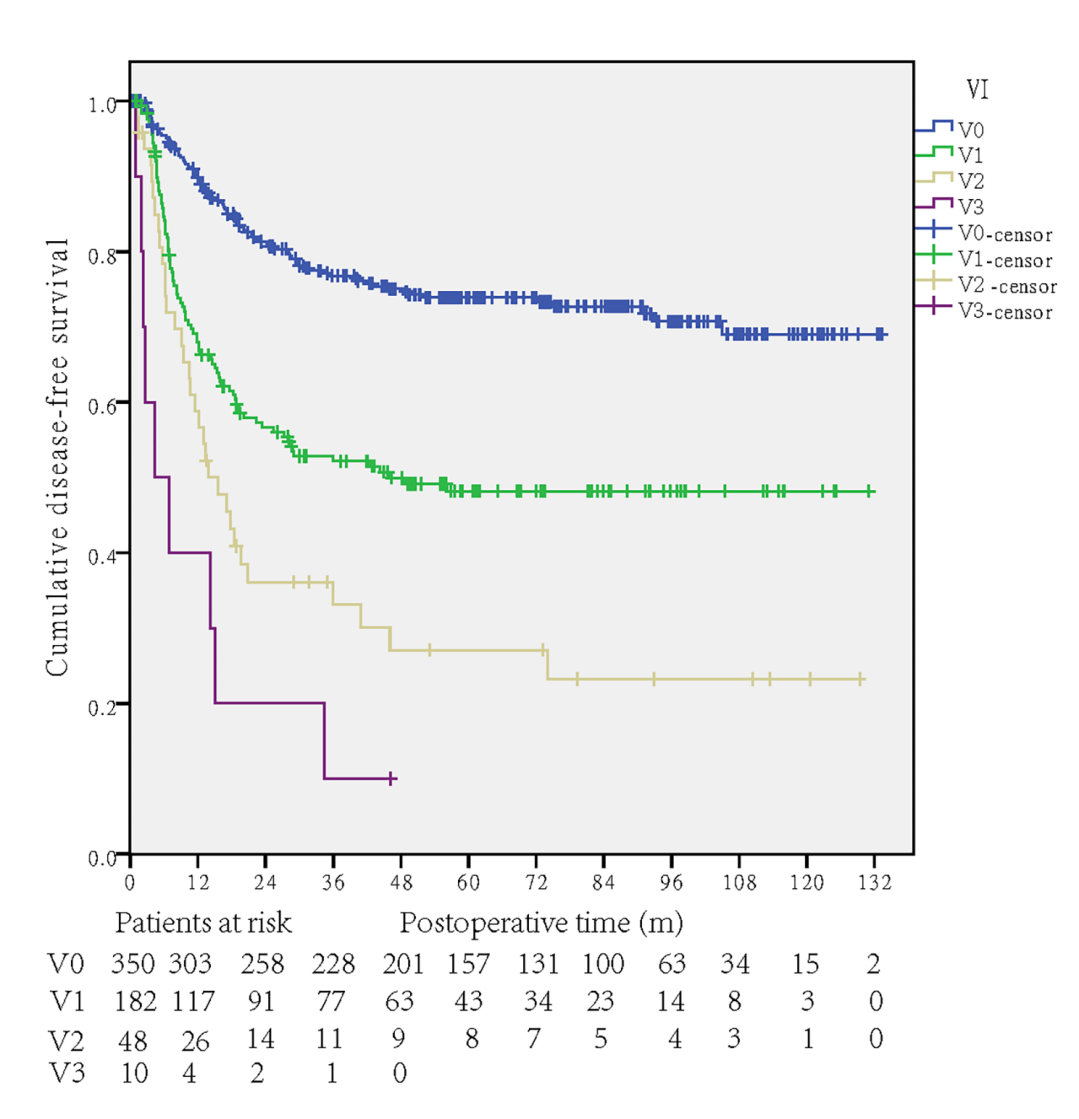
DFS curves of the degree of VI in univariate analysis

The 1-year, 3-year and 5-year disease-free survival rates were 79.7%, 64.7% and 61.2%, respectively. Univariate analysis demonstrated that age (p = 0.735), sex (p = 0.895), tumor location (p = 0.116), and tumor differentiation (p = 0.1) were not predictors of recurrence, while lymphatic invasion (p < 0.001), T category (p < 0.001), N category (p < 0.001), stage (p < 0.001) and the degree of venous invasion (p < 0.001, Fig. 2) were significant indicators of recurrence.

Multivariate analysis, the efficacy of which was determined using a chi-square test (χ2 = 246.05, p < 0.001), demonstrated that lymphatic invasion (HR: 1.457, 95% CI: 1.058–2.006, p = 0.021), T category (HR: 1.457, 95% CI: 1.058–2.006, p = 0.022), N category (HR: 1.535, 95% CI: 1.276–2.846, p < 0.001), stage (HR: 1.563, 95% CI: 1.235–1.976, p < 0.001) and the degree of venous invasion (HR: 1.526, 95% CI: 1.279–2.822, p < 0.001) were significant indicators for recurrence.

### The effect of venous invasion on recurrence stratified by pathological stage

**Fig. 3:**
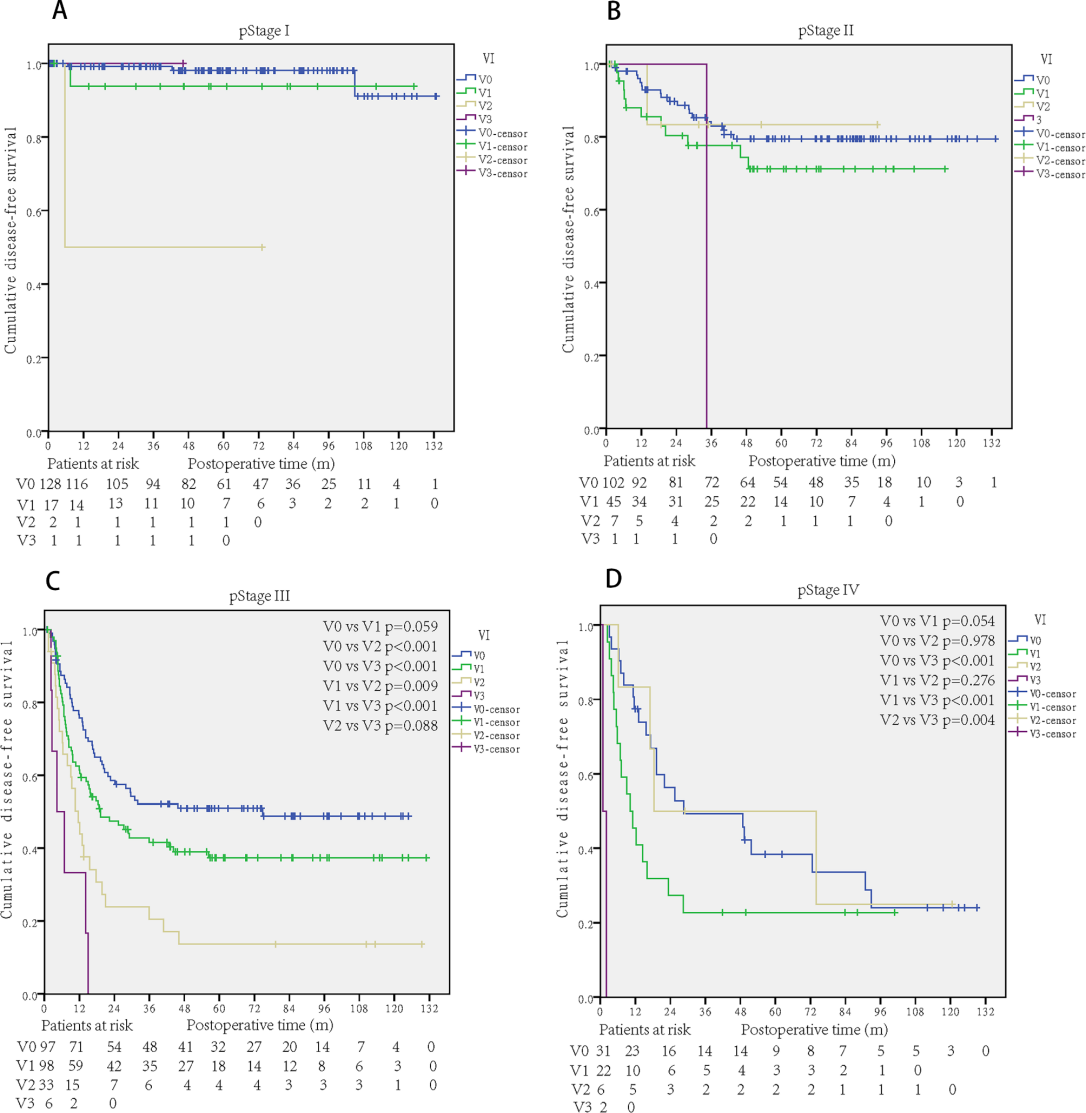
DFS curves of the degree of VI stratified by pathological stage. A: Stage I; B: Stage II; C: Stage III; D: Stage IV

The disease-free survival curves were distinguished well by the degree of venous invasion in stage III and IV patients (Fig. 3C and D). Limited by the case numbers of V2 and V3 in stages I and II, the curves were not well distinguished (Fig. 3A and B).

## Discussion

Our study aimed to explore a grading criterion for VI in ESCC patients, given the clinical significance between VI and ESCC demonstrated by the literature [7, 12–14]. The relationship between the grading of VI and lymphatic invasion, T category, N category and TNM stage was close. Lymphatic invasion, T category, N category, stage and grade of venous invasion were all poor indicators for DFS in univariable and multivariable analyses. A higher level of VI showed worse DFS. In the subgroup analysis stratified by pathological stage, DFS was well distinguished in stage III and IV patients.

The prognostic role of the level of VI has been well investigated in colorectal cancer. Shinto et al. studied the prognostic role of the number and size of venous invasions in pT3 colorectal cancer. An increase in the V-number can also lead to a worse prognosis [15]. Sato and his colleagues classified VI into three groups (G0, G1, G2) on the basis of the average number of VIs observed in a glass slide. The prognosis in colorectal cancer worsens as the VI grade increases [16]. Imai et al. divided VI into four groups (V0, V1, V2, V3) based on the number of VIs per glass slide. The results of his study suggested that V0 and V1 had a similar RFS in node-negative and well-to-moderately differentiated colorectal adenocarcinoma [17]. However, the grading criteria of VI in ESCC have not been clarified. Therefore, we established a method for the grading of VI on the basis of the number and size of Vis. The prognostic role of the level of VI in colorectal cancer is similar to the results of our study in ESCC. Our research indicated that DFS worsened from V0-V3 in univariable analysis in ESCC patients. The effect of venous invasion on recurrence stratified by pathological stage showed very good distinction in stage III and IV ESCC patients. The grading of VI was associated with lymphatic invasion, T category, N category and TNM stage.

The poor prognostic role of EMVI in colon cancer has been widely proved [18–21]. In addition, EMVI has been found to be an adverse prognostic parameter of survival for lymph node-negative colorectal cancer patients [22, 23]. EMVI is usually studied as an independent prognostic factor in colon cancer. However, few studies have focused on the effect of EMVI in esophageal cancer. Castonguay et al. reported that EMVI had no relationship with disease-specific survival in 103 esophageal adenocarcinoma patients [6]. Faiz et al. suggested that EMVI was a negative indicator for OS and DFS in EMVI+/N- locally advanced esophageal cancer patients [11]. Due to the limited number of studies and sample sizes of patients, we still classified EMVI within VI. Considering the potential prognostic value of EMVI, we assigned EMVI to V3 regardless of V-number and V-size in our research.

Some studies that concentrated on the clinical significance of VI adopted the H&E-staining method to identify VI [8, 14, 24, 25]. However, some studies adopted immunohistochemistry (IHC)-staining methods with CD-34 or Elastica van Gieson staining [6, 11, 13, 26, 27]. Although the positive rate of IHC in the same batch of patients was higher than that of H&E, the observation of VI could be easily accomplished on H&E-stained slides by most experienced pathologists [28]. VI was classified into IMVI and EMVI. EMVI identification in H&E-stained slides can be impeded due to obliterated vein muscular walls in specimens after surgery or due to destroyed vessel wall architecture and increased vessel fibrosis when patients receive neoadjuvant chemoradiotherapy [11]. Therefore, we excluded patients who received neoadjuvant chemotherapy (NAC). Since the impact of insufficiency of HE in identifying EMVI could be less with the exclusion of patients who received NAC, we adopted the H&E-staining method in the current study.

The survival curve of DFS showed good distinction in V0, V1, V2, and V3 in all patients regardless of stage. DFS worsened as the VI grade increased, especially in stage III and IV patients. The prognosis of ESCC patients with V3 was much worse than that of ESCC patients with V0 or V1. Lymphatic invasion may be an independent indicator for low-dose cisplatin plus 5-fluorouracil therapy in esophageal cancer patients after surgery [29]. Postoperative chemotherapy is usually recommended in stage III and IV ESCC patients. Almost all V3 patients with stage III and IV disease had recurrence within two years after surgery, which suggested that surgery and conventional postoperative adjuvant chemotherapy have encountered bottlenecks in these patients. Recently, the application of immunotherapy in esophageal cancer has received extensive attention. Adjuvant nivolumab for resected stage II and III esophageal cancer showed good survival benefits [30]. Powerful treatment strategies, such as immunotherapy after surgery or neoadjuvant immunotherapy, should be considered for stage III and IV ESCC patients with V3.

There are some limitations in our research. First, the routine method used to detect VI in our hospital was H&E, which is effective for most experienced pathologists. However, the most appropriate staining method involves Elastica van Gieson. Second, the number of patients with V3 in advanced stage ESCC patients was insufficient, which may have affected the results. We need to expand the sample size of ESCC patients.

In conclusion, our study explored an objective grading criterion of VI and proved the prognostic role of the degree of venous invasion in ESCC. The classification of venous invasion into 4 groups, namely, V0 (none), V1 (slight), V2 (moderate), and V3 (severe), is useful for the differentiation of prognosis in ESCC patients. The degree of VI in advanced ESCC patients for recurrence may have to be considered. However, large-sample studies with the IHC-staining method are needed. More research is needed to modify the grading criteria of VI in ESCC patients.

## Data Availability

All data generated or analysed during this study are included in this published article and its supplementary information files.
